# Adjuvant and immunostimulatory effects of a D-galactose-binding lectin from *Synadenium carinatum* latex (ScLL) in the mouse model of vaccination against neosporosis

**DOI:** 10.1186/1297-9716-43-76

**Published:** 2012-10-29

**Authors:** Mariana R D Cardoso, Caroline M Mota, Dâmaso P Ribeiro, Pablo G Noleto, William B F Andrade, Maria A Souza, Neide M Silva, Tiago W P Mineo, José R Mineo, Deise A O Silva

**Affiliations:** 1 Laboratory of Immunoparasitology, Institute of Biomedical Sciences, Federal University of Uberlandia, Av Pará 1720, Campus Umuarama, 38400-902, Uberlândia, MG, Brazil; 2 Laboratory of Molecular Biology, Institute of Biomedical Sciences, Federal University of Uberlândia, Uberlândia, MG, Brazil; 3 Laboratory of Immunopathology, Institute of Biomedical Sciences, Federal University of Uberlândia, Uberlândia, MG, Brazil

**Keywords:** *Neospora caninum*, Vaccination, Adjuvant, Plant lectin

## Abstract

Vaccination is an important control measure for neosporosis that is caused by a coccidian parasite, *Neospora caninum*, leading to abortion and reproductive disorders in cattle and serious economic impacts worldwide. A D-galactose-binding lectin from *Synadenium carinatum* latex (ScLL) was recently described by our group with potential immunostimulatory and adjuvant effects in the leishmaniasis model. In this study, we evaluated the adjuvant effect of ScLL in immunization of mice against neosporosis*.* First, we investigated in vitro cytokine production by dendritic cells stimulated with *Neospora* lysate antigen (NLA), ScLL or both. Each treatment induced TNF-α, IL-6, IL-10 and IL-12 production in a dose-dependent manner, with synergistic effect of NLA plus ScLL. Next, four groups of C57BL/6 mice were immunized with NLA + ScLL, NLA, ScLL or PBS. The kinetics of antibody response showed a predominance of IgG and IgG1 for NLA + ScLL group, whereas IgG2a response was similar between NLA + ScLL and NLA groups. Ex vivo cytokine production by mouse spleen cells showed the highest IFN-γ/IL-10 ratio in the presence of NLA stimulation for mice immunized with NLA + ScLL and the lowest for those immunized with ScLL alone. After parasite challenge, mice immunized with NLA + ScLL or ScLL alone presented higher survival rates (70-80%) and lower brain parasite burden as compared to PBS group, but with no significant changes in morbidity and inflammation scores. In conclusion, ScLL combined with NLA was able to change the cytokine profile induced by the antigen or lectin alone for a Th1-biased immune response, resulting in high protection of mice challenged with the parasite, but with low degree of inflammation. Both features may be important to prevent congenital neosporosis, since protection and low inflammatory response are necessary events to guide towards a successful pregnancy.

## Introduction

*Neospora caninum* is a coccidian parasite, closely related to *Toxoplasma gondii*, with broad host range and worldwide distribution, causing neuromuscular disease in dogs and abortion or reproductive disorders in cattle [1]. Neosporosis seriously impacts the dairy and beef industries leading to substantial economic losses [2]. Cattle acquire the infection by horizontal transmission, through ingestion of food and drinking water contaminated with oocysts, which are excreted in faeces of canine definitive hosts [3-5]; or more often, by transplacental vertical transmission, endogenous or exogenous, from an infected dam to her offspring during pregnancy [6]. Hence, tachyzoites can cross the placenta and infect the foetus, causing abortion or congenital infection, depending on the gestational age [7].

Humoral and cell-mediated immune responses are observed in experimentally and naturally infected cattle [8]. A Th1-type immune response characterized by increased production of IFN-γ, IL-12, and IgG2a isotype is usually protective against acute *N. caninum* infection in murine models [9]. However, this type of response may interfere with a successful pregnancy and lead to abortion if the infection occurs in early pregnancy [7]. On the other hand, a successful pregnancy depends on a switch to a Th2-type immune response at the maternal-fetal interface, in order to allow the acceptance of the fetal allograft [10]. In addition, innate immune response is also involved in host defense against *N. caninum* infection, mostly by means of Toll-like receptors (TLRs), such as TLR2 that participates in the induction of Th1-biased immune responses against this parasite [11].

Several control measures have been investigated to prevent *N. caninum* transmission and infection [2]. Numerous attempts to develop vaccines based on single or multiple recombinant proteins [12-15], proteins expressed from a vector system [16,17], γ-irradiated tachyzoites [18], attenuated tachyzoites [19], and different combinations of antigens and adjuvants [20-23], have been tested, but not yet with great success. All these vaccination strategies have shown that protection is sometimes partial and depends on the type of antigen as well as the adjuvant used.

Lectins are proteins that bind specifically to carbohydrates [24] and are isolated from different sources in nature. Plant lectins with increasing biological interest have been isolated from the Moraceae family, such as Jacalin and ArtinM from seeds of jackfruit (*Artocarpus integrifolia*), and have been widely described for its immunostimulatory potential as observed by robust cell proliferation and cytokine production [25-27]. A D-galactose-binding lectin named *Synadenium carinatum* latex lectin (ScLL) from the Euphorbiaceae family was recently described by our group [28] and its immunostimulatory and adjuvant effects have been determined in an experimental model of cutaneous leishmaniasis [29]. It was observed that ScLL associated or not with the soluble antigen from *Leishmania amazonensis* induced a potent Th1-biased immune response characterized by production of IL-12, IFN-γ, TNF-α mRNAs and specific IgG2a isotype, resulting in reduced infection rates after challenge with *L. amazonensis*[29]. In a model of asthma to study the effects of this lectin, it was observed that oral administration of ScLL inhibited neutrophil and eosinophil extravasations during acute and chronic inflammation, and reduced eosinophil and mononuclear blood counts during chronic asthma, showing an immunoregulatory effect [30].

Considering these relevant biological effects so far described for ScLL, the major aim of the present study was to evaluate if this lectin could exert any immunostimulatory and protective roles as adjuvant in an immunization protocol of mice for *Neospora caninum* infection.

## Materials and methods

### Parasite and antigen

*Neospora caninum* tachyzoites (Nc-1 isolate) [31] were maintained by serial passages in Vero cell line cultured in RPMI 1640 medium supplemented with 2% heat-inactivated calf fetal serum (CFS), 2 mM glutamine, 100 U/mL penicillin and 100 μg/mL streptomycin at 37°C in 5% CO_2_ atmosphere. Parasite suspensions were obtained as previously described [27]. Briefly, tachyzoites were harvested by scraping off the cell monolayer after 2–3 days of infection, passed through a 26-gauge needle and centrifuged at low speed (45 **×***g*) for 1 min at 4°C. The supernatant containing parasite suspension was collected, washed in phosphate buffered saline (PBS, pH 7.2), and the pellet was resuspended in PBS for antigen preparation or the tachyzoites were immediately used for animal challenge.

*Neospora* lysate antigen (NLA) was prepared as previously described [32]. Briefly, parasite suspension was treated with protease inhibitors and then lysed by freeze-thaw cycles followed by ultrasound on ice. After centrifugation (10,000 **×***g*, 30 min, 4°C), the supernatant was collected, filtered on 0.22 μm membranes and its protein concentration determined by bicinchoninic acid (BCA) assay [33]. NLA aliquots were stored at −20°C until using in immunization procedures, serological assays and in vitro and ex vivo cytokine production assays.

### Lectin from *Synadenium carinatum* latex (ScLL)

*Synadenium carinatum* specimens were harvested in Uberlândia, MG, Brazil, and registered in the Herbarium of the Federal University of Uberlândia (HUFU no. 84 38354). ScLL was obtained as previously described [28], with some modifications. Briefly, proteins were extracted from the fresh plant latex by gentle shaking with distilled water, at 1:10 proportion, for 48 h at 4°C. The mixture was centrifuged (3500 **×***g*, 30 min, 4°C) and the supernatant was filtered on 0.45 μm membranes, consisting in the total aqueous extract. Next, the total extract was submitted to affinity chromatography using immobilized D-galactose column on agarose (Pierce, Rockford, IL), equilibrated with 5 mM borate-buffered saline, pH 8.0 (BBS). The D-galactose-binding lectin (ScLL) was eluted with 0.4 M D-galactose (Sigma Chemical Co., St Louis, MO) in BBS, dialyzed against deionized water and the protein concentration was determined by BCA assay [33]. Hemagglutination assays were performed to confirm the lectin activity of ScLL as previously described [28]. ScLL aliquots were stored at −20°C until using in immunization procedures and in vitro cytokine production assays.

### Animals

Female C57BL/6 mice with 8–12 weeks old were obtained from the School of Medicine of Ribeirão Preto (FMRP), University of São Paulo (USP), Ribeirão Preto, SP, Brazil. Animals were maintained under standard conditions in the Animal Facility from Federal University of Uberlândia (UFU), Uberlândia, MG, Brazil. All procedures were conducted according to guidelines for animal ethics and the study received approval of the Ethics Committee for Animal Experimentation of the Institution.

### In vitro cytokine production assays

Naive C57BL/6 mice (n=3) were euthanized, their thigh-bone and shin-bone were removed and bone marrow cells were aseptically collected for cell culture. Bone marrow cells were differentiated in dendritic cells as previously described [34], by treating cells with 30 ng/mL of granulocyte-macrophage colony-stimulating factor (GM-CSF) in RPMI medium supplemented with 20% CFS for 7 days. Afterward, cells were washed with RPMI medium containing 10% CFS continuously in an ice bath for removal of differentiated dendritic cells. Cell suspensions were washed (400 × *g*, 10 min, 4°C) and the pellet was resuspended in RPMI medium containing 10% CFS. Viable cells were counted in a hemocytometer, using the Trypan blue exclusion vital stain, with more than 90% cell viability.

Dendritic cell suspensions (2 × 10^5^ cells/200 μL/well) were cultured in triplicate in 96-well culture plates and stimulated with NLA at different concentrations (25, 10, 5, and 1 μg/mL), or ScLL (10, 1, 0.1, and 0.01 μg/mL) in RPMI medium with 10% CFS. In further experiments, cells were stimulated with NLA at 25 μg/mL plus ScLL at different concentrations (10, 1, 0.1, and 0.01 μg/mL), since the highest concentration of NLA showed to have better stimulatory effect. Cells stimulated with medium alone or LPS (10 μg/mL) were included as controls. Cells were incubated for 48 h at 37°C in 5% CO_2._ Cell supernatants were then collected, centrifuged (3000 **×***g*, 5 min, 4°C) and stored at −70°C for the detection of TNF-α, IL-6, IL-10 and IL-12p40. Cytokine measurements were performed by sandwich ELISAs according to manufacturer’s instructions (R&D Systems, Minneapolis, MN). The limit of detection was 31 pg/mL for all cytokines.

### Immunization procedures

Four groups of 13 mice were immunized subcutaneously three times at two-week intervals, with 200 μL/animal of the following formulations: 25 μg of NLA plus 50 μg of ScLL in sterile PBS (NLA + ScLL group); 25 μg of NLA alone (NLA group); 50 μg of ScLL alone (ScLL group); and PBS only. The adopted doses of antigen and lectins were based on previous studies [27,29]. Blood samples were collected at 0, 15, 30, 45, 60 and 90 days after immunization (d.a.i.), and the sera were analyzed for the presence of specific antibodies. Two weeks after the last immunization (45 d.a.i.), three mice in each group were euthanized and their spleens were aseptically removed for cell culture and cytokine production assay.

### Ex vivo cytokine production assays

Spleens were macerated in RPMI medium and cell suspensions were washed with medium, treated with lysis buffer (0.16 M NH_4_Cl and 0.17 M Tris–HCl, pH 7.5), washed again and resuspended in RPMI medium containing 10% CFS. Cells (2 × 10^5^ cells/200 μL/well) were cultured in triplicate in 96-well culture plates in the presence of mitogen (Concanavalin A - ConA, 2.5 μg/mL), antigen (NLA, 10 μg/mL) or medium alone and incubated at 37°C in 5% CO_2_ atmosphere. After 48 h, the cell-free supernatants were collected and stored at −70°C for cytokine quantification. IL-10 and IFN-γ measurements were performed by sandwich ELISAs according to manufacturer’s instructions (R&D Systems, Minneapolis, MN). The limit of detection was 31 pg/mL for both cytokines.

### Challenge of immunized mice

Four weeks after the last immunization (60 d.a.i.), ten animals per group were challenged intraperitoneally with 2×10^7^ Nc-1 tachyzoites. Animals were observed daily for clinical signs, by evaluating morbidity scores, body weight changes and mortality. Morbidity scores were calculated as previously described [35], with modifications: sleek/glossy coat, bright and active (score 0); ruffled coat (score 1); hunched, tottering gait, starry stiff coat (score 2), reluctance to move (score 3). All surviving animals were euthanized at 30 days after challenge (90 d.a.i.), when brain tissues were collected, sliced longitudinally, being half stored at −70°C for quantification of parasite burden by real-time polymerase chain reaction (PCR) assay target “Nc-5”. The remaining tissue was fixed in 10% buffered formalin, embedded in paraffin and routinely processed for immunohistochemical and histological assays.

### Determination of *N. caninum*-specific antibodies

Levels of *N. caninum*-specific total IgG, IgG1 and IgG2a antibodies were measured by ELISA as previously described [21,27]. Briefly, high-binding microtiter plates were coated with NLA (0.5 μg/well) and blocked with 5% skim milk in PBS plus 0.05% Tween 20 (PBS-T). Serum samples were diluted 1:25 in 1% skim milk-PBS-T and incubated for 1 h (IgG) or 2 h (IgG1 and IgG2a) at 37°C. After washing, peroxidase-labeled goat anti-mouse IgG (1:1000; Sigma) or biotin-labeled goat anti-mouse IgG1 (1:4000) or anti-mouse IgG2a (1: 2000) antibodies (Caltag Lab. Inc., South San Francisco, CA) were added and incubated for 1 h at 37°C. Next, streptavidin-peroxidase (1:1000; Sigma) was added for IgG1 and IgG2a detection assays. The assays were developed with 0.01 M 2,2-azino-bis-3-ethyl-benzthiazoline sulfonic acid (ABTS; Sigma) and 0.03% H_2_O_2_. Optical density (OD) values were determined in a plate reader at 405 nm.

### Determination of parasite burden in brain tissue

Parasite burden in the brain tissue from all surviving mice after 30 days of challenge was determined by quantitative real-time polymerase chain reaction (qPCR) as described elsewhere [21]. The following typical Nc-5 primers (Np6/Np21) were used: sense (3’-GCTGAACACCGTATGTCGTAAA-5’) and antisense (3’-AGAGGAATGCCACATAGAAGC-5’) to detect the *N. caninum* Nc-5 sequence through SYBR green detection system (Invitrogen, San Francisco, CA). DNA extraction was performed from 20 mg of murine brain tissues using the Wizard SV Genomic DNA kit (Promega Co., Madison, WI) according to the manufacturer’s instructions. DNA concentrations were determined by UV spectrophotometry (260 nm) and adjusted to 200 ng/μL with sterile DNAse free water. Assays to determine *N. caninum* tachyzoite loads were performed through real-time PCR (7500 Real time PCR System, Applied Biosystems, Foster City, CA) and parasite burden was calculated by interpolation from a standard curve with DNA equivalents extracted from Nc-1 tachyzoites included in each run. Brain tissue from non-immunized and unchallenged mice was analyzed in parallel as negative control.

Immunohistochemical assays were also performed to verify brain tissue parasitism as previously described [21,27]. Briefly, deparaffinized sections were blocked with 3% H_2_O_2_, treated with 0.2 M citrate buffer (pH 6.0) in microwave oven to rescue antigenic sites, and blocked with 2% non-immune goat serum. Next, primary antibody consisting of sera from mice experimentally infected with *N. caninum* was added followed by secondary biotinylated goat anti-mouse IgG antibody (Sigma) and avidin–biotin peroxidase complex (ABC kit, PK-4000; Vector Laboratories Inc., Burlingame, CA). The reaction was developed with 0.03% H_2_O_2_ plus 3,3Â´-diaminobenzidine tetrahydrochloride (DAB; Sigma) and slides were counterstained with Harris haematoxylin and examined under light microscopy.

### Determination of inflammation scores in brain tissue

Brain tissue sections obtained from all surviving mice after 30 days of challenge were stained with haematoxylin and eosin, according to previously described protocol [21,27], and then examined by microscopy to detect tissue damage. Inflammation scores were represented as arbitrary units: 0–1, mild; 1–2, moderate; 2–3, severe and > 3, very severe. Brain tissue from non-immunized and unchallenged mice served as negative controls.

### Statistical analysis

Statistical analysis was carried out using GraphPad Prism 5.0 (GraphPad Software Inc., San Diego, CA). Differences between groups were analyzed using ANOVA or Kruskal-Wallis test, when appropriate, with the respective Bonferroni or Dunn multiple comparison post-tests to examine all possible pairwise comparisons. Student *t* test was used for comparison between IgG1 and IgG2a isotypes in different groups. The Kaplan–Meier method was applied to estimate the percentage of mice surviving at each time point after challenge and survival curves were compared using the Log-rank test. Values of *P* < 0.05 were considered statistically significant.

## Results

### In vitro cytokine production by dendritic cells stimulated with NLA and/or ScLL

**Figure 1:**
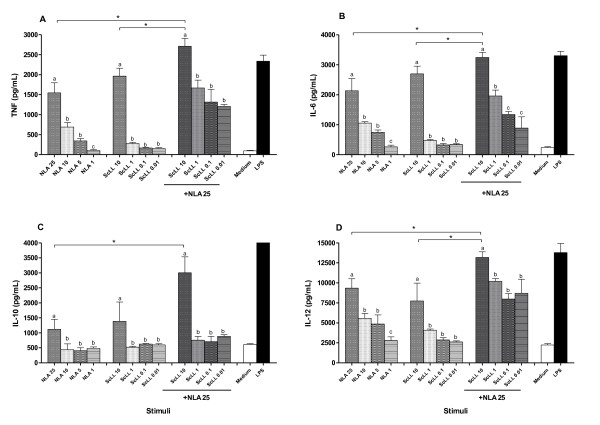
**In vitro cytokine production by bone marrow-derived dendritic cells from naive C57BL/6 mice.** Cells were stimulated with *Neospora* lysate antigen (NLA) at different concentrations (25, 10, 5 and 1 μg/mL), or *Synadenium carinatum* latex lectin (ScLL) at different concentrations (10, 1, 0.1 and 0.01 μg/mL) or NLA (25 μg/mL) plus ScLL (10, 1, 0.1 and 0.01 μg/mL). Cells stimulated with medium alone or LPS were included as controls. Cell-free supernatants were collected after 48 h and analyzed for detection of TNF-α **(A)**, IL-6 **(B)**, IL-10 **(C)** and IL-12 **(D)** by ELISA. Values are indicated as mean ± SD of cytokine levels in pg/mL. ^a-c^ Different letters indicate statistically significant differences between the concentrations in each group analyzed; *Statistically significant differences for the highest concentrations among groups (ANOVA and Bonferroni multiple comparison post-test; *P* < 0.05).

Bone marrow-derived dendritic cells from naive C57BL/6 mice were stimulated with NLA and/or ScLL at different concentrations for cytokine assays in cell supernatants after 48 h of treatment (Figure 1). When cells were stimulated with NLA alone, it was observed a dose-dependent response for TNF-α (Figure 1A), IL-6 (Figure 1B), IL-10 (Figure 1C) and IL-12 (Figure 1D), with the higher cytokine levels detected for the greatest concentration of NLA (25 μg/mL). The ScLL lectin alone or associated with NLA was also able to induce higher cytokine levels for the greatest concentration of ScLL (10 μg/mL) in relation to other concentrations (*P* < 0.05). When the three groups of stimulus were compared regarding the greatest concentration of each stimulus, NLA associated with ScLL induced higher levels of all cytokines than NLA or ScLL alone (*P* < 0.05), except for IL-10 in comparison with ScLL alone (Figure 1C). Also, NLA associated with ScLL induced cytokine levels similar to the LPS stimulus.

### Humoral immune response after immunization and parasite challenge

**Figure 2:**
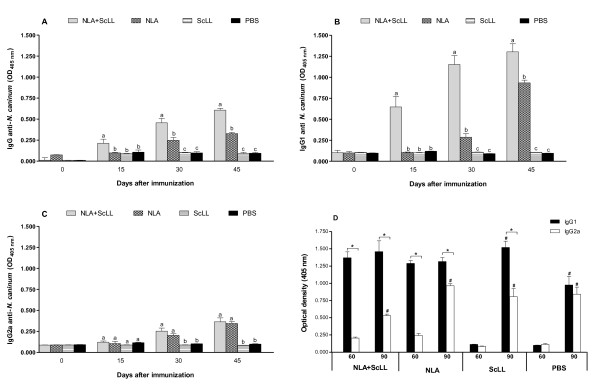
**Levels of IgG (A), IgG1 (B) and IgG2a (C) anti-*Neospora caninum *determined by ELISA in sera of C57BL/6 mice immunized subcutaneously three times (0, 15, and 30 days) with *Neospora* lysate antigen (NLA) associated with *Synadenium carinatum *latex lectin (ScLL).** As controls, mice were inoculated with NLA alone (antigen control), ScLL alone (lectin control) or PBS (infection control). Values are indicated as optical density (OD) at 405 nm and expressed as mean ± SEM. ^a-c^ Different letters indicate statistically significant differences between groups for each time point analyzed (ANOVA and Bonferroni multiple comparison post-test; *P* < 0.05). **(D)** Comparison between IgG1 and IgG2a levels to *Neospora caninum* determined at 60 (before challenge) and 90 (after challenge) days after immunization. Mice were challenged with 2x10^7^ tachyzoites of the Nc-1 isolate at 60 days after immunization. *Statistically significant differences between IgG1 and IgG2a in each time point analyzed; ^#^Statistically significant differences when compared to the time before challenge (60 days after immunization) for each antibody isotype within each group (Student *t* test; *P* < 0.05).

The kinetics of IgG, IgG1 and IgG2a antibody responses to *N. caninum* was determined by ELISA in sera of C57BL/6 mice after immunization with NLA and/or ScLL lectin or PBS (control group) (Figure 2). Mice immunized with NLA + ScLL showed higher levels of IgG anti-*N. caninum* from 15 days after immunization (d.a.i.) until the 45^th^ d.a.i. in relation to NLA, ScLL or PBS groups (*P* < 0.05) (Figure 2A). IgG production by mice immunized with NLA alone was only detected from the 30^th^ d.a.i. onwards (Figure 2A). A similar kinetics was observed for IgG1 anti-*N. caninum*, with NLA + ScLL group presenting higher levels of this isotype from 15 to 45 d.a.i. as compared to other groups and NLA group showing detectable IgG1 levels from the 30^th^ d.a.i. (*P* < 0.05) (Figure 2B). A different pattern was found for IgG2a anti-*N. caninum*, with NLA + ScLL group showing similar levels of this isotype in relation to NLA group from 30 to 45 d.a.i. (Figure 2C).

Seroconversion of control groups and comparison between IgG1 and IgG2a levels to *N. caninum* were analyzed before (60 d.a.i.) and after (90 d.a.i.) parasite challenge in all experimental groups (Figure 2D). Before challenge, all animals immunized with NLA alone or associated with ScLL presented higher levels of IgG1 than IgG2a (*P* < 0.05). After challenge, this predominant IgG1 pattern was maintained, although no significant increase in IgG1 levels was observed after challenge, except for the ScLL and PBS control groups, thus confirming their seroconversion. In contrast, IgG2a levels were significantly increased after challenge in all experimental groups, with similar levels between IgG1 and IgG2a only in the PBS group (Figure 2D).

### Ex vivo cytokine production by spleen cells from mice after immunization

**Figure 3:**
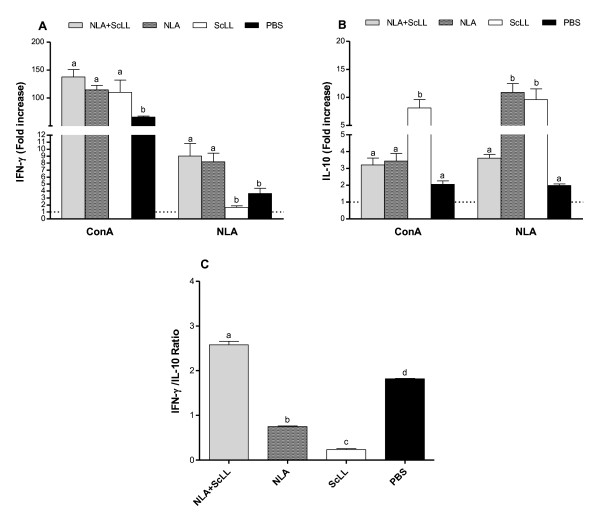
**Ex vivo cytokine production by spleen cells from mice immunized with *Neospora *lysate antigen (NLA) associated with *Synadenium carinatum *latex lectin (ScLL).** As controls, mice were inoculated with NLA alone (antigen control), ScLL alone (lectin control) or PBS (infection control). Spleen was collected from three mice per group at 45 days after immunization and cells were cultured in the presence of mitogen (Concanavalin A [ConA] 2.5 μg/mL), antigen (NLA, 10 μg/mL) or medium alone. Cell-free supernatants were collected after 48 h and analyzed for detection of IFN-γ **(A)** and IL-10 **(B)** by ELISAs. The IFN-γ/IL-10 ratio was calculated only for NLA stimulation **(C)**. Values are indicated as mean ± SEM of cytokine levels in relation to baseline (medium) and the dashed line represents the baseline. ^a-d^ Different letters indicate statistically significant differences for each time point analyzed (ANOVA and Bonferroni multiple comparison post-test; *P* < 0.05).

Two weeks after the last booster of immunization (45 d.a.i.), three mice per group were euthanized and their spleens were aseptically collected for cytokine assays. After antigen stimulation, IFN-γ production was higher in NLA + ScLL and NLA groups in comparison to ScLL and PBS groups (*P* < 0.05), even though no significant difference was observed between the former groups (Figure 3A). IL-10 was detected in higher concentration in the NLA and ScLL groups in relation to the other groups (*P* < 0.05) (Figure 3B). The IFN-γ/IL-10 ratio was also calculated (Figure 3C) and results showed a higher ratio for the NLA + ScLL group and a lower ratio for the ScLL group in comparison to the other groups (*P* < 0.05).

After mitogen stimulation, all groups showed cytokine production above the baseline (medium), markedly for IFN-γ in the groups NLA + ScLL, NLA and ScLL (Figure 3A), whereas IL-10 production was moderate and more evident in the group with ScLL alone (Figure 3B).

### Mortality and parasite burden after challenge

**Figure 4:**
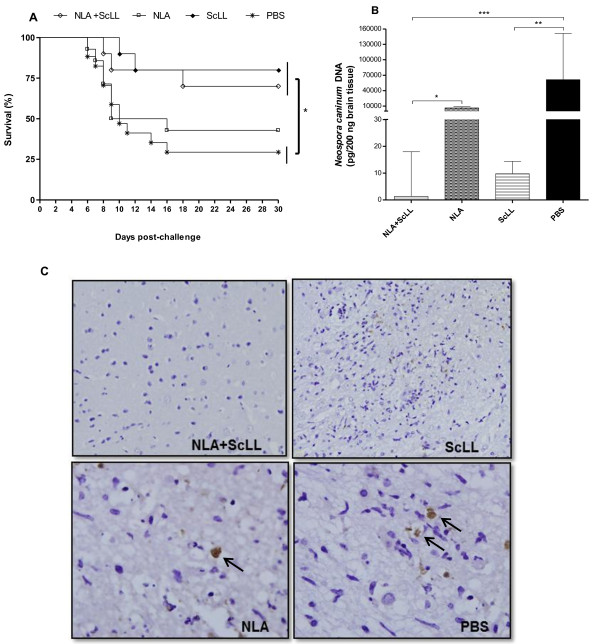
**(A) Survival curves of C57BL/6 mice after challenge with *Neospora caninum*.** Four mouse groups (13 animals per group) were immunized with either *Neospora* lysate antigen (NLA) associated with *Synadenium carinatum* latex lectin (ScLL), or NLA alone (antigen control), or ScLL alone (lectin control), or PBS (infection control). Mice (10 animals per group) were challenged with 2x10^7^ tachyzoites of the Nc-1 isolate at 60 days after immunization. *P < 0.05 (Log-rank test). **(B)** Parasite burden in the brain tissue from all surviving mice after 30 days of challenge and analyzed by real-time PCR. Bars represent median with interquartile range. *P < 0.05; **P < 0.01; ***P < 0.001 (Kruskal-Wallis test and Dunn multiple comparison post-test). **(C)** Representative photomicrographs of immunohistochemical assays in the brain tissues from mice of the four groups: NLA + ScLL, NLA, ScLL, and PBS. Arrows indicate strongly stained free tachyzoites and parasitophorous vacuoles from *N. caninum* in NLA and PBS panels (original magnification,×100); NLA + ScLL and ScLL panels (original magnification, × 40).

Four weeks after the last immunization (60 d.a.i.), animals were challenged with a lethal dose of Nc-1 tachyzoites and were monitored for 30 days for the evaluation of morbidity scores, body weight changes and mortality. At the end of this experimental phase, all surviving animals were euthanized for evaluation of brain parasite burden by qPCR. The animals of all groups showed clinical signs starting from 2–4 days after challenge and reached morbidity maximum scores on 6–8 days after challenge. There were no significant differences in morbidity scores and body weight changes among all groups (Additional file 1: Figure S1). On the other hand, after 30 days of challenge, the higher survival rates were found for the groups NLA + ScLL (70%) and ScLL (80%), showing survival curves significantly different from the PBS group (29%) (*P* < 0.05) (Figure 4A). In parallel, parasite load in the central nervous system was found to be lower in NLA + ScLL and ScLL groups in relation to PBS group (*P* < 0.05) as determined by qPCR (Figure 4B). Immunohistochemical assays for detection of brain tissue parasitism confirmed qPCR results, since a lower parasitism was observed in the NLA + ScLL and ScLL groups in relation to the NLA group and mostly to the PBS group that showed strongly stained *N. caninum* tachyzoites and parasitophorous vacuoles (Figure 4C). Negative controls of the reaction included brain tissue sections incubated with non-immune mouse serum or in the absence of primary antibodies, and no staining was observed (data not shown).

### Brain tissue inflammation after parasite challenge

**Figure 5:**
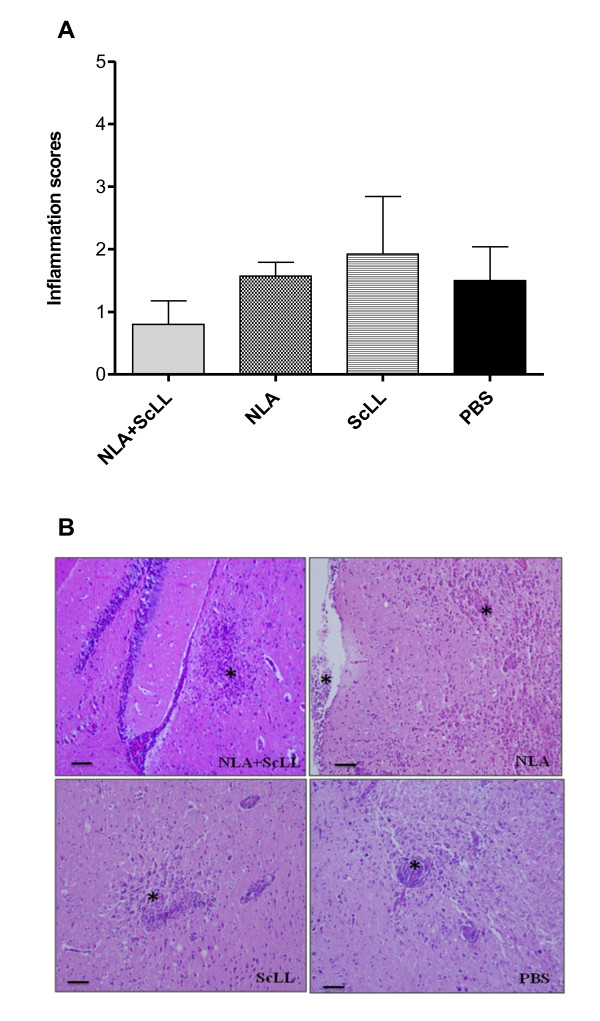
**(A) Inflammatory scores in brain tissues from all surviving C57BL/6 mice after *Neospora caninum* challenge.** Four groups of mice (13 animals per group) were immunized with either *Neospora* lysate antigen (NLA) associated with *Synadenium carinatum* latex lectin (ScLL), or NLA alone (antigen control), or ScLL alone (lectin control), or PBS (infection control). Mice (10 animals per group) were challenged with 2x10^7^ tachyzoites of the Nc-1 isolate at 60 days after immunization and histopathological changes were analyzed. Bars represent mean ± SEM. **(B)** Representative photomicrographs of histological assays in brain tissues from mice of the four groups: NLA + ScLL, NLA, ScLL and PBS. Asterisks (*) indicate mononucleated cell infiltrates and/or infiltrates in the meninges. Haemtoxylin and eosin staining. Bar scale: 50 μm.

After 30 days of challenge, histological analysis of brain tissue was performed to determine inflammatory scores. Although the NLA + ScLL group showed the lowest inflammatory score and the ScLL group the highest, no statistical significance was found among the groups (Figure 5A). The histological changes were characterized by mononucleated cell infiltrates organized in glial nodules, presence of perivascular cuffing by lymphocytes and focal mononucleated cell infiltrates in the meninges. Representative photomicrographs of each group are illustrated in Figure 5B.

## Discussion

Adjuvants play an important role in the efficacy of vaccines, contributing to increase the potency and kinetics of the immune response and to direct the type of this response. In the induction of immune responses, protein-carbohydrate interactions are essential since they represent cell-to-cell contacts, leading to crosstalk among cells and consequently their activation [36]. Lectins can play this role given that it may bind carbohydrates and function as pattern recognition receptors (PRRs) in host cells [36]. Several plant lectins have shown adjuvant or immunostimulatory effects in different infections [25,27,29,37]. Among them, the lectin from the *S. carinatum* latex (ScLL) has been recently studied for its peculiar roles in different experimental models, having an adjuvant effect in cutaneous leishmaniasis [29] or immunoregulatory action in asthma murine models [30].

Given that currently there is no effective treatment for neosporosis, control methods are necessary. Among them, the removal of infected animals is the only way to reduce incidence of the disease in herds. However, given the high prevalence of *N. caninum* and the low occurrence of abortion in herds (less than 5% of cows abort), it does not make sense to cull infected animals. Usually animals are culled due to poor reproductive success, which *Neospora* infection may be one reason. Thus, vaccination may be a viable method of reducing the incidence of neosporosis and improving the reproductive performance in herds [2]. In this context, several studies have been conducted to obtain reliable formulations in vaccination strategies in order to protect against *N. caninum* infection [38-40], but studies using plant lectins as adjuvants in neosporosis are scarce in the literature. Recently, we evaluated the role of lectins (ArtinM and Jacalin) from the jackfruit (*Artocarpus integrifolia*) and showed that ArtinM, a D-mannose binding lectin, presented stronger immunostimulatory and adjuvant effect than Jacalin, a D-galactose binding lectin, in immunization of mice against neosporosis, by inducing a protective Th1-biased pro-inflammatory immune response and higher protection after parasite challenge [27].

In the present study, we investigated if another D-galactose binding lectin, ScLL obtained from the *Synadenium carinatum* latex, could play immunostimulatory role and adjuvant effect in mouse immunization against *N. caninum* infection as already reported in other infection models*.* First, we evaluated the in vitro cytokine production by dendritic cells stimulated with antigen, lectin or by the combination of both. We found that NLA, ScLL or NLA + ScLL produced the highest levels of all cytokines investigated (TNF-α, IL-6, IL-10 and IL-12), showing a typical profile of a dose-dependent response, when tested in their greatest concentrations. Also, it was clearly evident the synergistic effect of NLA and ScLL, since cells stimulated with NLA + ScLL secreted the highest levels of all cytokines. These findings indicate that ScLL was able to increase the immunogenicity of the antigen, demonstrating its adjuvant role in the in vitro experiments. In addition, as dendritic cells are professional APCs and have critical functions in modulation of the primary immune response to intracellular parasites, this can be particularly important in vaccination strategies for *N. caninum*. In this context, the TLR2 innate immune receptor is decisive for dendritic cell activation during *N. caninum* infection, by using the TLR2/MyD88 recognition pathway with posterior expression of costimulatory and antigen-presentation molecules, T lymphocyte programming, and pro-inflammatory cytokine production, associated with an appropriate IgG synthesis against the parasite [11]. Furthermore, ScLL itself has already shown to increase the expression of IL-12, IL-1 and TNF-α mRNAs in macrophages pretreated with the lectin (10 μg/mL) and infected with *L. amazonensis*[41].

The kinetics of the humoral immune response after immunization of mice with NLA, ScLL or both revealed a distinct profile between the IgG1 and IgG2a isotypes, with predominance of IgG1 in all time points analyzed for the NLA + ScLL group, thus reflecting its higher production of IgG in relation to other groups. Before parasite challenge (60 d.a.i.), the NLA group reached similar IgG1 levels to the NLA + ScLL group, but significantly higher than IgG2a levels, demonstrating that NLA alone is also able to induce a Th2-biased humoral immune response. After parasite challenge, there was no significant increase in the levels of IgG1 in NLA-immunized groups, even though they maintained higher than IgG2a in all experimental groups. Regarding the control groups, seroconversion was observed in response to challenge with predominance of the IgG1 isotype, particularly evident in the ScLL group, indicating that this lectin alone is able to direct a Th2-biased humoral immune response. In contrast, IgG2a levels increased after parasite challenge in all groups, and this may represent a shift from Th2-biased immune response to a more protective Th1-biased immune response. Our previous study also showed higher levels of IgG1 than IgG2a after challenge in all groups of mice immunized with NLA associated or not with lectins ArtinM or Jacalin, indicating that this Th2 type of humoral immune response seems to be dependent on the antigen rather than the adjuvant [27]. Indeed, our previous study has shown a considerable increment in both antibody isotypes after challenge in NLA-immunized groups associated or not with CpG, suggesting that the parasite was able to induce both Th2 and Th1 immune responses, although a Th2-biased humoral response was more evident [21].

In order to observe the immunization-induced cytokine profile before parasite challenge, we evaluated the ex vivo cytokine production by spleen cells from mice immunized with NLA associated or not with ScLL. After antigenic stimulation, both NLA + ScLL and NLA groups showed similar and increased IFN-γ production, but a distinct pattern was found for IL-10, with increased cytokine production in the groups immunized with antigen or lectin alone. When the IFN-γ/IL-10 ratio was analyzed in an attempt to verify the balance between pro-inflammatory and anti-inflammatory cytokines, the highest ratio was found for the NLA + ScLL group and the lowest for the ScLL alone. These results suggest that the ScLL lectin alone is able to induce higher levels of IL-10, leading to an anti-inflammatory or regulatory immune response pattern. It is noteworthy that spleen cells from the ScLL group stimulated with mitogen also showed the highest IL-10 production. In our previous study [27] mice immunized with NLA alone also showed high IL-10 levels, reinforcing the role of the NLA antigen in inducing an anti-inflammatory or immunoregulatory response. In addition, mice immunized with NLA plus Jacalin or Jacalin alone exhibited the lowest ratio IFN-γ/IL-10 ratio whereas those immunized with NLA + ArtinM or ArtinM alone presented the highest ratio, suggesting that the lectin adjuvant, but not the antigen, was able to modulate the cytokine production, leading to a Th1 type-biased pro-inflammatory immune response [25,27,37].

In the present study, the combination of NLA plus ScLL was also able to change the cytokine profile induced by the antigen or lectin alone for a Th1-biased immune response, indicating a potential adjuvant effect. In our previous studies, however, the ScLL lectin has shown contrasting roles depending on the study model. In immunization of mice against *L. amazonensis*, ScLL induced increased levels of IgG2a and overexpression of IL-12 and TNF-α mRNAs, leading to a Th1-biased immune response that supported the protective function of this lectin against cutaneous leishmaniasis [29]. Primed macrophages when treated with ScLL showed no significant nitrite oxide (NO) production after infection with *L. amazonensis*, although they were able to reduce parasite intracellular proliferation [41]. Additionally, oral administration of ScLL reduced IL-4 and IL-5 levels in murine models of acute and chronic inflammation and lead to increased IFN-γ and IL-10 production in asthma inflammatory model, directing a shift from Th2- to Th1-biased immune response [30].

It is well known that a protective immune response against *N. caninum* should be a non-exacerbated Th1-type response [2]. Accordingly, in a previous study we demonstrated that a strong cellular immune response associated with increased IFN-γ production and inflammation induced by vaccination with *N. caninum* excreted-secreted antigen (NcESA) alone or CpG-adjuvanted NcESA rendered mice more susceptible to parasite challenge [21]. In the present study, mice immunized with NLA plus ScLL or ScLL alone presented the highest survival rate (70-80%) and the lowest brain parasite burden, with no significant differences in morbidity scores and body weight changes from baseline. Therefore, the ScLL lectin associated or not with NLA is able to confer protection against parasite challenge regardless of inflammation. Accordingly, a previous study suggests that ScLL inhibits IκBα degradation, a negative regulator of pro-inflammatory NF-κB [30]. In our previous study, mice immunized with ArtinM combined with NLA presented a higher survival rate (86%), even though with high brain tissue inflammation scores, whereas animals immunized with Jacalin alone or associated with NLA had the lowest inflammation scores, but with intermediate survival rates (50–62%) [27]. Therefore, the induction of a pro-inflammatory immune response by ArtinM plus NLA seems to be more favorable than detrimental to the host to control neosporosis. This has been reported for the control of leishmaniasis, where ArtinM plus soluble *Leishmania* antigen induced a potent pro-inflammatory response with reduction of the parasite load, but without decreasing the lesion size [25]. In the present study, however, mice immunized with NLA alone presented a low survival rate associated with a high brain parasite burden, similarly to the PBS control group, suggesting that NLA alone stimulated a non-protective Th2-biased immune response [21].

Recent studies have shown that different administration routes and delivery strategies in vaccination against neosporosis in murine models may play a key role in directing the immune response towards a more protective pattern [39,42,43]. In this framework, innate responses are crucial for stimulating the Th1-type antibody-and cell-mediated immune responses and lectins play an important role in the protein-carbohydrate binding that mediates the parasite-host cell interactions.

Altogether, the results of the present study showed that ScLL, a D-galactose-binding lectin, when combined with NLA antigen can be used as powerful adjuvant in immunization procedures against neosporosis, resulting in high protection of mice challenged with the parasite, but with low degree of inflammation. In addition, the D-galactose-binding seems not be essential for these effects, since another D-galactose binding lectin, Jacalin, showed to be a poor adjuvant in immunization against neosporosis [27]. Future studies should be conducted to evaluate if the immunostimulatory and adjuvant effects of the ScLL lectin may be important to prevent congenital neosporosis, since protection and low inflammatory response are necessary events to guide towards a successful pregnancy.

## Competing interests

The authors declare that they have no competing interests.

## Authors’ contributions

MRDC was a MS student and was involved in the lectin preparation, mouse immunization procedures and parasite challenge, cytokine and antibody assays, statistical analysis, and preparation of the draft manuscript. CMM and DPR were MS students and were involved in the parasite maintenance in cell culture, antigen preparation, mouse immunization procedures and parasite challenge, and determination of brain parasite load by qPCR. PGN and WBF were undergraduate students and were involved in the lectin preparation, care with the animals and daily observation for clinical signs and mortality. MAS was a PhD collaborator and was involved in the lectin preparation protocols. NMS was a PhD collaborator and was involved in the immunohistochemical and histopathological assays. TWPM and JRM were PhD collaborators and were involved in the experimental design, data analysis and revision of the manuscript. DAOS was a PhD researcher and was involved in the study design, interpretation of results, statistical analysis and had final responsibility for the study. All authors read and approved the manuscript.

## Supplementary Material

Additional file 1 **Figure S1.** Morbidity score (A) and body weight change from baseline (B) curves of C57BL/6 mice after challenge with Neospora caninum. Four mouse groups (13 animals per group) were immunized with either Neospora lysate antigen (NLA) associated with Synadenium carinatum latex lectin (ScLL), or NLA alone (antigen control), or ScLL alone (lectin control), or PBS (infection control). Mice (10 animals per group) were challenged with 2x10^7^ tachyzoites of the Nc-1 isolate at 60 days after immunization and observed daily up to 30 days after challenge. Data are expressed as mean ± SEM in surviving mice. There were no significant differences in morbidity scores and body weight changes among all groups. Click here for file
