# Supplementary material for: Adjuvant and immunostimulatory effects of a D-galactose-binding lectin from Synadenium carinatum latex (ScLL) in the mouse model of vaccination against neosporosis
Source: Vet Res. 2012 Oct 29;43(1):76. doi: 10.1186/1297-9716-43-76 (PMC3583070; doi:10.1186/1297-9716-43-76)
Supplement: Supplementary file 1 [file 1297-9716-43-76-S1.doc]

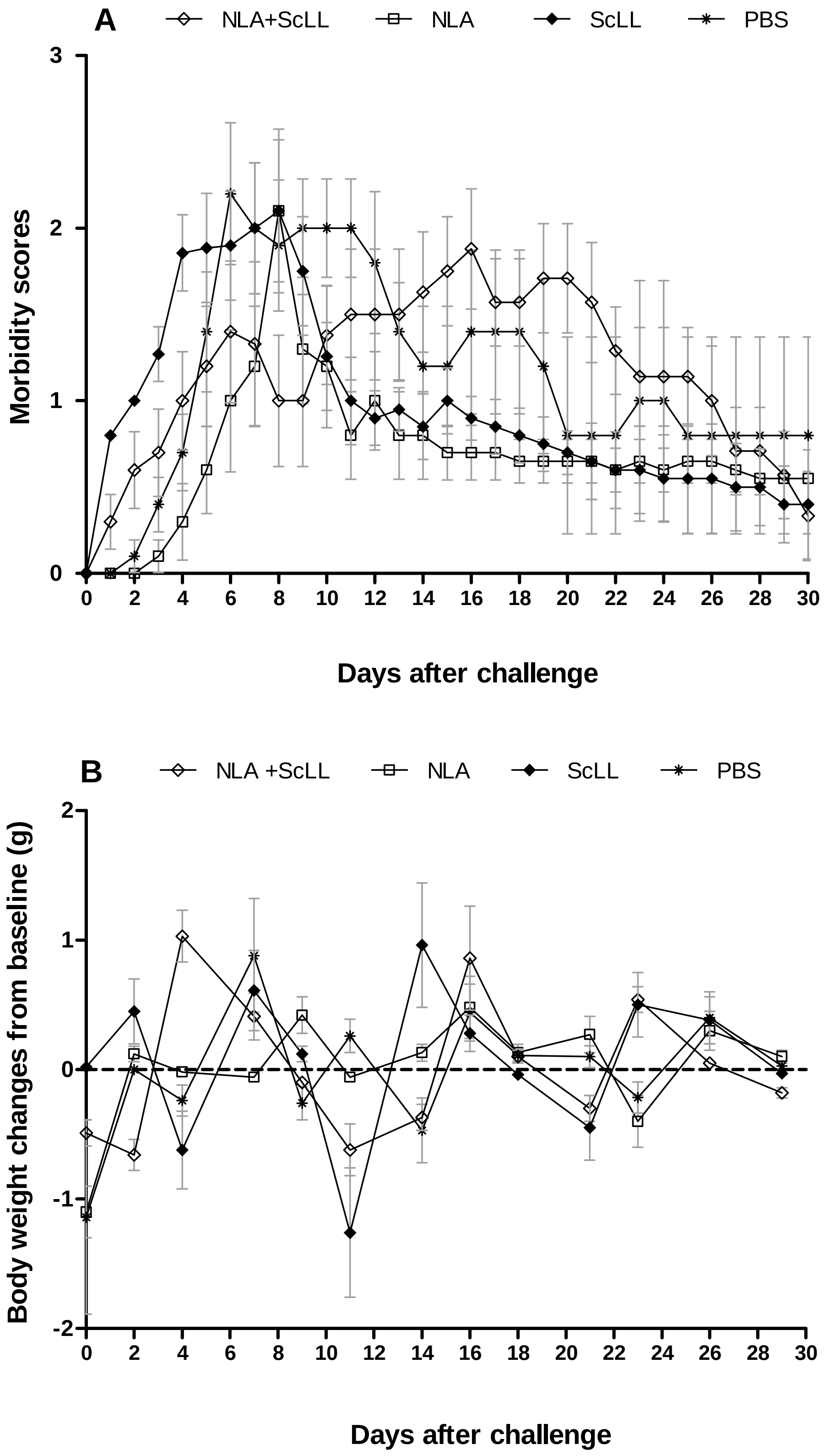
A
NLA+ScLL
NLA
ScLL
PBS
Morbidity scores
Days after challenge
B
NLA +ScLL
NLA
ScLL
PBS
Body weight changes from baseline (g)
Days after challenge
